# Internal High Echoes Can Suggest the Possible Resection of Ovarian Vein Leiomyosarcoma: A Case Report

**DOI:** 10.1002/iju5.70025

**Published:** 2025-05-09

**Authors:** Sohei Iwagami, Shoji Oura, Masaya Nishihata

**Affiliations:** ^1^ Department of Urology Kishiwada Tokushukai Hospital Kishiwada Japan; ^2^ Department of Surgery Kishiwada Tokushukai Hospital Kishiwada Japan

**Keywords:** EUS, internal high echoes, leiomyosarcoma, ovarian vein, resectability

## Abstract

**Introduction:**

Leiomyosarcomas of ovarian veins often affect the IVC, the duodenum, and kidneys. Therefore, the resectability of the leiomyosarcomas highly depends on the presence or extent of their invasion to the surrounding organs.

**Case Presentation:**

A 55‐year‐old woman with a retroperitoneal mass on CT was referred to our hospital. CT and MRI showed a lobulated large mass with broad contact with the duodenum. EUS showed focal internal high echoes in the tumor areas adjacent to the duodenum. After obtaining the pathological diagnosis of leiomyosarcoma, the tumor was successfully removed without duodenum resection. Post‐operative pathological findings confirmed the absence of direct leiomyosarcoma invasion to the duodenum and the sparse atypical cells with edematous background in the high internal echo areas.

**Conclusions:**

Surgeons should note that internal high echoes in the tumor areas adjacent to the duodenum can be an important predictor of possible resection of the retroperitoneal tumors.

**Abbreviations**
CT: computed tomography
EUS: endoscopic ultrasonography
FNA: fine needle aspiration
IVC: inferior vena cava
MRI: magnetic resonance imaging

**Summary**

- Ovarian vein leiomyosarcomas are rare disorders and are often detected as large tumors with invasion to the surrounding organs.
- Internal high echoes in the tumor areas adjacent to the duodenum on EUS can predict no invasion of the tumor to the duodenum.

## 1 Introduction

Leiomyosarcomas are highly aggressive disorders and are sometimes observed in the retroperitoneum [1]. They are the second most common retroperitoneal tumors in adults and highly originate from the IVC, but rarely from ovarian veins [2, 3].

Resectability of retroperitoneal tumors, especially those around the right kidney, mainly depends on whether the tumors invade the IVC or the duodenum. When the tumors spread extensively into the IVC, tumor resection sometimes needs a cardiopulmonary bypass. In addition, duodenal invasion of the retroperitoneal tumors naturally needs pancreaticoduodenectomy. It, therefore, is crucial for surgeons to judge whether the retroperitoneal tumors invade the IVC or the duodenum and to determine the extent of invasion when judged as positive for invasion.

We herein report a case of large ovarian vein leiomyosarcoma, which was assessed as resectable based on the preoperative imaging findings.

## 2 Case Presentation

**FIGURE 1:**
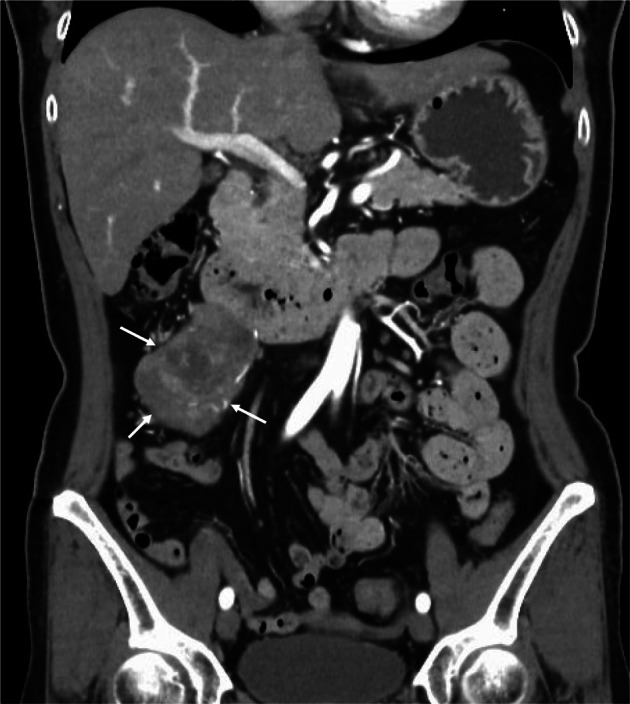
CT findings. CT showed a polygonal tumor with heterogeneous enhancement (arrows) adjacent to the duodenum.

**FIGURE 2:**
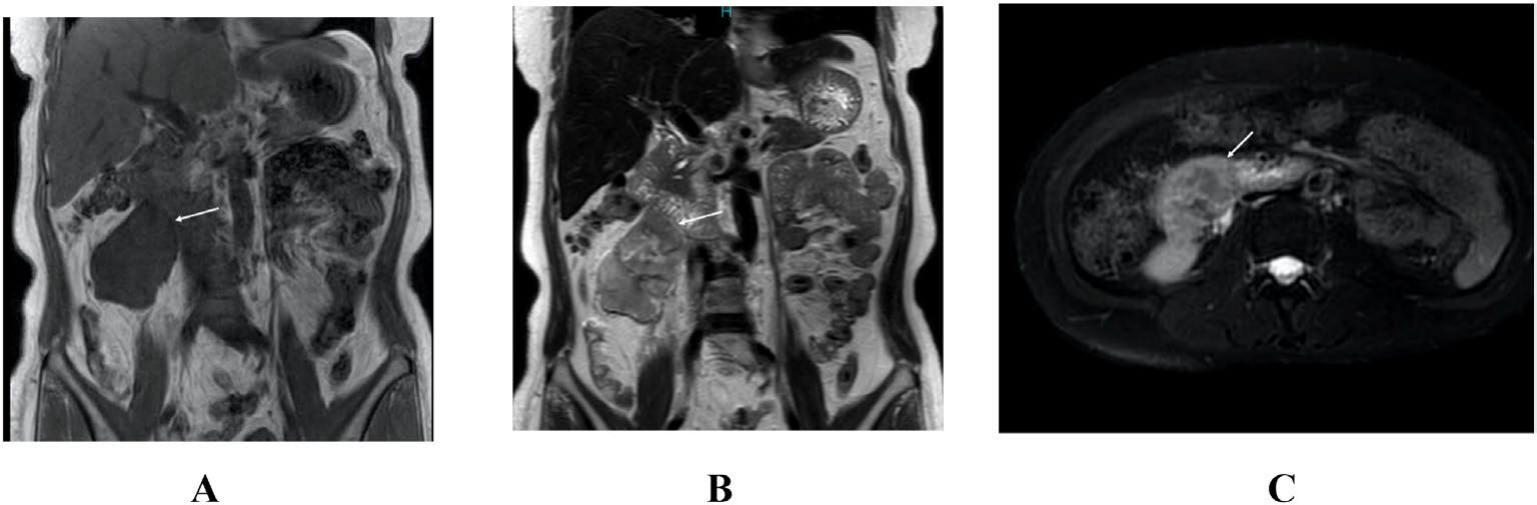
MRI findings. (A) The tumor showed a hypointense pattern on T1‐weighted coronal images with unclear findings of the duodenal invasion (arrow). (B) A demarcation hyper intense line (arrow) was observed between the tumor and the duodenum on T2‐weighted coronal images, but could not deny the invasion of the tumor into the duodenum. (C) Fat‐suppressed T2‐weighted axial images showed a hyperintense pattern in the tumor areas adjacent to the duodenum.

**FIGURE 3:**
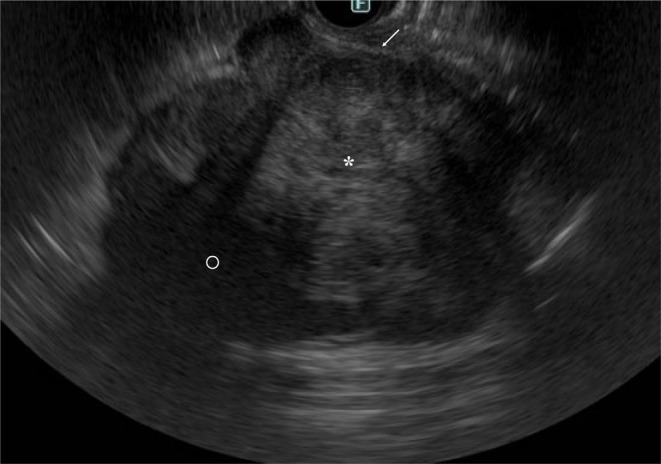
EUS findings. EUS showed a non‐interrupted white line (arrow) between the tumor and the duodenum, and focal high internal echoes (asterisk) and very low internal echoes (open circle) in the proximal and distal parts of the tumor to the duodenum, respectively.

**FIGURE 4:**
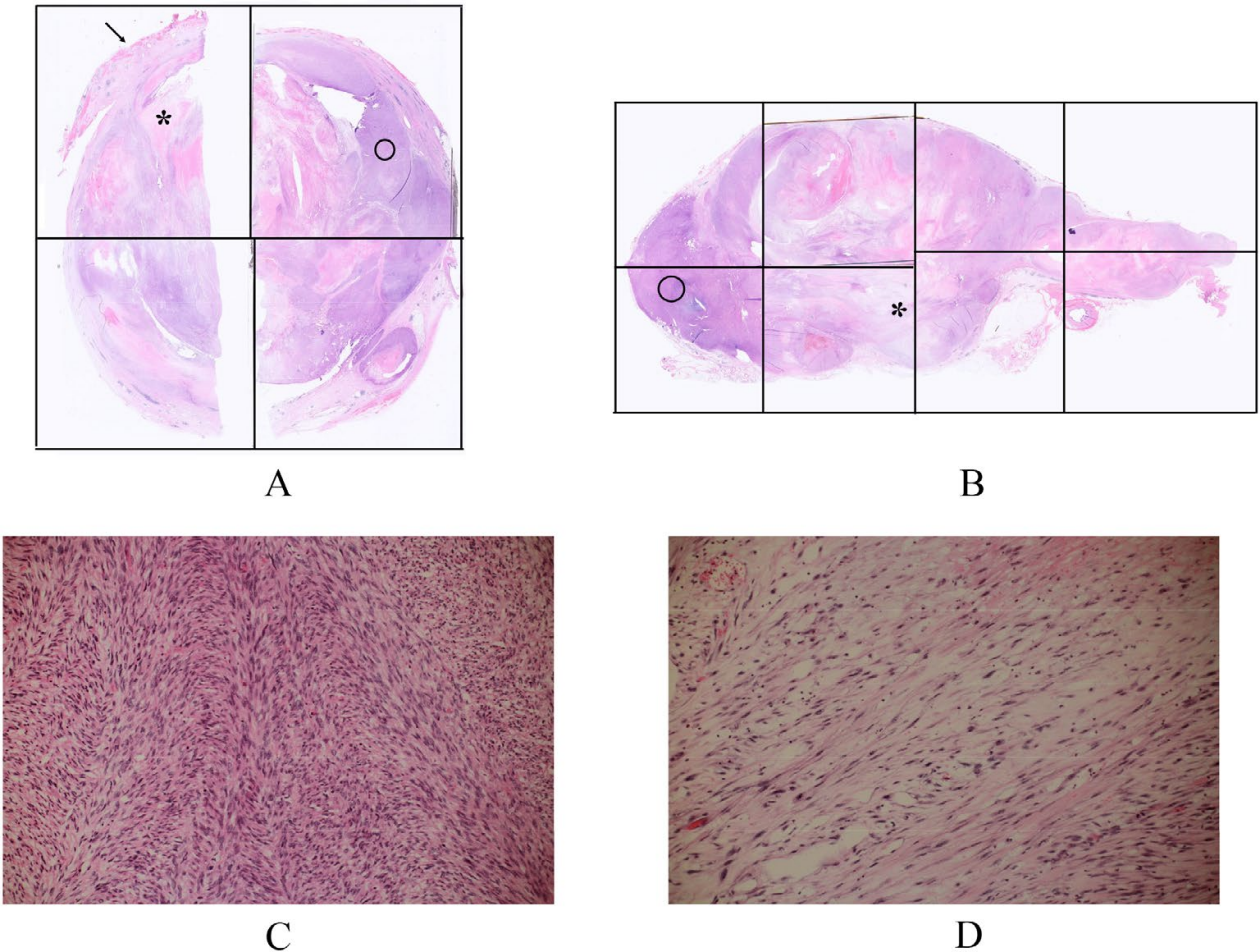
Pathological findings. (A) Low magnified short‐axis tumor combined view showed no malignant cell invasion to the duodenal adventitia (arrow), edematous areas (asterisk), and spindle cell‐rich areas (open circle). (B) Low magnified long‐axis tumor combined view showed edematous areas (asterisk) and spindle cell‐rich areas (open circle). Magnified view of the spindle cell‐rich areas showed dense spindle cells growing in a complex fashion (H.E. × 100). (C) Magnified view of the edematous areas showed sparse spindle cells in the edematous background (H.E. × 100).

A 55‐year‐old woman with abdominal pain was referred to our hospital. CT showed an irregularly enhanced mass, 59 mm in short axis size, which was in close contact with the right ureter, had suspected invasion to the IVC, and had extensive contact with the descending to horizontal duodenum (Figure 1). Ultrasound showed a lobulated mass with distinct borders, internal low echoes, and focal internal high echoes. MRI of the tumor showed a hypo‐intense pattern on T1‐weighted images, a mosaic pattern on T2‐weighted images, and a hyper‐intense line at the duodenal borders on T2‐weighted images (Figure 2). On EUS, the tumor was a well‐circumscribed lobulated mass with intact duodenal mucosae, no clear continuity between the mass and the duodenal wall, and at least focal internal high echoes at the tumor areas adjacent to the duodenum (Figure 3). FNA biopsy using a 22G needle, done three times, showed atypical spindle cells growing in a complicated fashion with SMA positivity and DOG1, S100, and CD34 negativities on immunostaining, leading to the diagnosis of ovarian vein leiomyosarcoma. The patient, therefore, underwent tumor resection combined with a right nephroureterectomy. Surgical procedures around the IVC could be done without any problems, followed by successful dissection between the duodenum and the tumor, though there was some hard adhesion at the biopsy sites, by additive resection of the adventitia of the duodenum. Post‐operative pathological study showed a lobulated mass, 110 mm in long‐axis size, with focal hemorrhage/necrosis areas and negative surgical margins. Atypical cells grew in a cord‐like fashion and had spindle‐shaped nuclei both with eosinophilic cytoplasm and varying degrees of pleomorphism. Internal high and low echo areas had sparse atypical cells with edematous background and densely packed atypical cells, respectively (Figure 4). Immunostaining of the resected tumor showed similar immunostaining results to those of the biopsy specimen concerning SMA, DOG1, S100 protein, and CD34 and a high Ki‐67 labeling index of 45%. The patient recovered uneventfully and was discharged on the 11th day after surgery. The patient received postoperative adjuvant radiation therapy to the operative field and has been well for 3 months.

## 3 Discussion

The presence and extent of invasion to the large vessels and the duodenum markedly affect the surgical outcome of retroperitoneal tumors. We did not find any difficulties in the surgical procedures around the IVC areas and the nephroureterectomy in this case. Preoperative image, especially MRI, findings suggested possible management of the duodenum. It, however, was difficult for us to completely rule out the tumor invasion to the duodenum. Non‐epithelial solid malignancies had heterogeneous internal structures as the tumors grew larger [4, 5]. In fact, this large retroperitoneal tumor had heterogeneous structures on all image modalities. However, due to the extensive contact between the tumor and the duodenum, neither CT nor MRI could present definitive information about the duodenal invasion to us.

The backscattering of ultrasound waves defines the internal echoes of solid masses [6, 7]. Similarities of acoustic impedances in the relatively uniform cell areas generate low internal echoes due to the paucity of ultrasound wave backscattering. Conversely, it is well known that high internal echoes can be generated by the massive presence of papillary/septal structures, the interminglement of pathological components with markedly different acoustic impedances such as fat and water, or the existence of an edematous background with sparse cell components [8].

Massive papillary/septal structures are very rarely seen in leiomyosarcomas. In addition, fat‐suppressed T2‐weighted images clearly ruled out the possible presence of fat components and diffuse distribution of microcysts in the tumor. Therefore, an edematous background with sparse cell components, that is, less aggressive pathological findings, seemed best to explain the mechanisms of focal internal high echoes in the tumor areas adjacent to the duodenum. Pathological findings actually supported this mechanism for the internal high echo formation.

The vast majority of non‐epithelial malignant tumors are accompanied by the proliferation of spindle cells, being particularly prominent in areas with high proliferative potential [9]. The densely proliferated cells with similar acoustic impedance generally generate low internal echoes. In other words, internal high echoes on EUS highly suggest the possible resection of the tumor due to the suspected low proliferative ability, at least in the areas adjacent to the duodenum.

Spanish group for research in sarcomas highly recommends that oncologists resect the EUS biopsy route to prevent local recurrence by tumor seeding [10]. In this case, possible resection was predicted by preoperative imaging findings and made the clinical implication of EUS biopsy less important. However, even though the possibility of a pancreaticoduodenectomy was very low, we needed to demonstrate malignant cells in order to obtain the patient's consent for surgery. The EUS biopsy puncture sites are planned to be followed up with regular endoscopic examinations and, upon any abnormalities at the biopsy sites, to be treated with endoscopic treatment whenever possible.

## 4 Conclusions

Internal high echoes adjacent to the duodenum on EUS can be a predictive factor for the possible resection of retroperitoneal tumors. It, therefore, seemed imperative that oncologists pay close attention to the internal echoes of the tumor, especially in the areas contacting the duodenum on EUS when assessing the resectability of retroperitoneal tumors.

## Consent

Written informed consent was obtained from the patient for the publication of this case.

## Conflicts of Interest

The authors declare no conflicts of interest.
